# Recent HIV infection among women and men enrolling to care in urban clinics in Ethiopia

**DOI:** 10.1186/s12879-026-13443-y

**Published:** 2026-04-30

**Authors:** Ilili Jemal Abdulahi, Per Björkman, Sviataslau Sasinovich, Dawit Hailu Alemayehu, Dawit Arimide, Adane Mihret, Alemseged Abdissa, Andargachew Mulu, Patrik Medstrand, Anton Reepalu

**Affiliations:** 1 Department of Translational Medicine, Lund University, J Waldenströms gata 35, Malmö, 20502 Sweden; 2 Armauer Hansen Research Institute, Addis Ababa, Ethiopia; 3 Department of Infectious Diseases, Skåne University Hospital, Malmö, Sweden; 4 Department of Clinical Microbiology, University and Regional Laboratories, Lund, Sweden

**Keywords:** HIV, Recent Infection, Viral load, Key population, Ethiopia

## Abstract

**Background:**

Early HIV diagnosis allows timely interventions to control HIV transmission. We have determined the proportion of recent infections among people living with HIV (PLHIV) enrolling to care in Ethiopia and identified factors associated with recent infection.

**Methods:**

Participants (aged ≥ 15 years) newly enrolled in HIV care were recruited from urban clinics in central Ethiopia (2022–2024). We used a recent infection testing algorithm, combining limiting antigen avidity serology and viral load quantification, to determine HIV infection recency. Factors associated with recent infection were investigated using logistic regression analysis, including sex-stratified analyses.

**Results:**

Among 622 participants (median age 35 years; 364 [58.5%] women), 42 (6.8%) had recent infection. Recent infection was more common among PLHIV aged 15–24 years compared to those > 24 years (adjusted odds ratio [aOR], 4.3; 95% confidence interval [CI], 2.1–8.6), and in PLHIV belonging to key and priority populations (aOR, 2.2; 95% CI, 1.1–4.2). In sex-stratified analyses, age 15–24 years remained significantly associated with recent infection in both men (unadjusted odds ratio, 6.4; 95% CI, 1.5–26.5) and women (aOR, 2.7; 95% CI,1.2–6.1), whereas belonging to key and priority populations was significantly associated with recent infection only among women (aOR, 2.4; 95% CI, 1.1–5.2).

**Conclusion:**

A low proportion of PLHIV newly enrolled in care had recently acquired HIV infection. Recent infection was more common among persons aged 15–24 years and women belonging to key and priority populations. Further scale-up of HIV testing services are needed to improve detection of recent infection, which could help in prevention of new infections.

## Background

Despite decreasing HIV incidence in recent decades in sub-Saharan Africa, 50% of an estimated 1.3 million new infections reported globally in 2024 occurred in this region. These figures illustrate that current efforts remain insufficient to meet the 2030 Sustainable Development Goal of ending AIDS [1, 2]. To achieve this goal, it is important to identify people living with HIV (PLHIV) early after acquisition of infection, to enable time-sensitive interventions that may reduce HIV transmission, especially rapid initiation of antiretroviral therapy (ART) and contact tracing [3]. People with recently acquired HIV infection have higher risk of onward HIV transmission due to high-level viremia as well as ongoing risk behavior [4–7]. Understanding the magnitude of recent infections provides critical insights into transmission dynamics and can help guide effective prevention.

Several approaches have been used to identify recent HIV infections. Serological testing is a cross-sectional method for determining recency, which has become widely used [8, 9]. However, serological assays have limitations since their performance is influenced by factors such as ART use and HIV subtype [8, 10]. The World Health Organization (WHO) therefore recommends combining serologic recency assays with viral load (VL) quantification for determination of HIV infection recency to reduce misclassification and to improve accuracy [9]. The overall adult (15–49 years) HIV prevalence in Ethiopia, home to the second largest population in sub-Saharan Africa, is estimated at 0.67% [11], which is low compared to many other countries in this world region. However, the Ethiopian HIV epidemic is concentrated mainly to urban areas and to certain key and priority populations (KPP) (especially female sex workers) [12]. Of the few studies from Ethiopia that have examined recency of HIV infection, none included viral load in determining recency, thus solely relying on serological assays [13, 14]. Hence, data on magnitude of recent infections in this country remains largely unknown.

We aimed to determine the proportion of recent HIV infection among PLHIV newly enrolled in HIV care at urban ART clinics in central Ethiopia. In addition, we have investigated characteristics of men and women newly enrolling in HIV care, with particular focus on those with recent infection at presentation to care. Furthermore, in sex-stratified analysis, we analyzed whether factors associated with recent infection differed for men and women.

## Methods

This nested cross-sectional study was conducted by selecting eligible participants from a prospective cohort study on HIV transmission in central Ethiopia, including PLHIV aged 15 years or older (Transmission of HIV in the Era of Scaling up Access to Antiretroviral Therapy [THESA], ClinicalTrials.gov NCT05652400, registered on December 15, 2022). The study was conducted in a mainly urban area in and around the city of Adama in the central Oromia region of Ethiopia. The Ethiopian HIV epidemic is highly heterogeneous. According to 2025 estimates from the Ethiopian Public Health Institute, this area has > 1% incidence and 5% prevalence for HIV among persons aged 15–49 years, respectively [11]. From the THESA cohort study participants, we selected newly enrolled PLHIV with HIV diagnosis date of ≤1 week before inclusion into the cohort study. Participants were enrolled June 2022 to April 2024.

### Laboratory methods

At study inclusion, venous blood was collected in EDTA tubes, followed by centrifugation and separation of plasma, which was aliquoted and stored at −80 °C at a regional study laboratory. Plasma aliquots were subsequently shipped to the Clinical Virology Laboratory, Lund University, Sweden. The Maxim HIV-1 Limiting Antigen Avidity (Maxim Biomedical, Inc., USA) assay was used to determine recency of HIV infection, following the manufacturer’s instructions. In brief, samples with an initial singular run normalized optical density (ODn) value ≤ 2 were retested in triplicate to confirm the ODn values [15]. HIV-1 VL was determined (for participants with ODn value of ≤1.5) at the Department of Clinical Microbiology, Region Skåne Medical service, Sweden, using Cobas 5800 System and at the Clinical Virology Laboratory, Lund University, using an in-house RT-qPCR assay [16–18]. Viral load results were used with ODn values for the final classification of recency of HIV infection. Both VL and limiting antigen (LAg) avidity results were managed using the LAg data management file provided by the manufacturer.

### Study definitions

Preliminary recent HIV infection was defined as ODn value ≤ 1.5 in the Maxim HIV-1 LAg avidity test. Confirmed recent HIV infection was based on LAg avidity results (ODn ≤ 1.5) in combination with HIV RNA > 1000 copies/mL, in line with the recent infection testing algorithm (RITA) [9, 19, 20]. All newly enrolled participants who did not meet the criteria for confirmed recent infection were classified as having non-recent infection. Persons identified as recent infections were estimated to have acquired HIV infection in the preceding six months [15].

For this study, we considered individuals as belonging to KPP using the World Health Organization guidelines and the Ethiopian national strategic plan definition, including the following occupations: commercial sex worker (CSW), occupational driver (including long distance driver), prisoner, military/police and daily laborer [12, 21–23]. Participants with no permanent residence and those living with relatives or friends were categorized as having an unstable living situation, while those who owned or rented a house or apartment were categorized as having a stable living situation.

### Statistical analysis

Data was analyzed using SPSS version 30 (IBM Corp., Armonk, NY, USA). Median with interquartile range was used for continuous variables. Frequency and percentage were computed for categorical variables. The distribution of socio-demographic and clinical characteristics was compared between male and female study participants by applying Chi-square test.

We determined the proportion of recent and non-recent HIV infection among PLHIV newly enrolled in care. Ninety-five percent confidence intervals (CI) were computed using the Wilson score interval. Characteristics of people with recent HIV infection were compared to participants with non-recent HIV infection using chi-square and Mann-Whitney test for categorical and continuous variables, respectively.

Multivariable logistic regression analysis was carried out to identify factors associated with recent HIV infections. Variables with *p* < 0.25 in univariate analysis were included in a multivariable model followed by stepwise removal of the least significant variable until only variables with *p* < 0.05 remained in the final model. Furthermore, to assess potential effect modification by sex, we included interaction terms between sex and selected variables (age, population group, and housing condition) in the logistic regression model. Additionally, sex-stratified analysis was conducted to explore potential sex-associated differences.

## Results

### Study participants

A total of 622 participants newly enrolled in HIV care were included in this study. The majority were female (*n* = 364, 58.5%). Youth (15–24 years) comprised 12.4% (77/622) of the participants. Notably, 17.3% of female participants were youth (63/364), compared to 5.4% of male participants (14/258). Female participants were younger than male participants (median age 32 vs 38 years). A higher proportion of male participants belonged to KPP compared to female (56.2% vs 36.8%). Daily labourers accounted for the largest proportion of KPP in both male (*n* = 140; 75.9%) and female (*n* = 93; 69.4%) participants, followed by occupational drivers for male (*n* = 28; 16.6%) and CSW (*n* = 38; 28.4%) for female participants. Socio-demographic characteristics differed between male and female participants (Table 1).

**Table 1:** Characteristics of people living with HIV newly enrolled at urban ART clinics by sex, central Ethiopia

Characteristic	Overall (*n* = 622)	Male (*n* = 258)	Female (*n* = 364)	*p*-Value
Age (years; median, IQR)	35 (28–43)	38 (31–45)	32 (26–40)	
Age				**<0.001**
15–24 years	77 (12.4%)	14 (5.4%)	63 (17.3%)	
25–49 years	466 (74.9%)	201(77.9%)	265 (72.8%)	
>50 years	79 (12.7%)	43 (16.7%)	36 (9.9%)	
Marital status				**<0.001**
Single	162 (26.1%)	70 (27.2%)	92 (25.3%)	
Married	241 (38.8%)	131 (51.0%)	110 (30.2%)	
Divorced	144 (23.2%)	45 (17.5%)	99 (27.2%)	
Widowed	74 (11.9%)	11 (4.3%)	63 (17.3%)	
Residence				0.110
Urban	547 (87.9%)	220 (85.3%)	327 (89.8%)	
Rural	75 (12.1%)	38 (14.7%)	37 (10.2%)	
Education				**0.020**
Illiterate	162 (26.1%)	51 (19.8%)	111 (30.6%)	
<6 grades	142 (22.9%)	66 (25.6%)	76 (20.9%)	
6–12 grades	276 (44.4%)	123 (47.7%)	153 (42.1%)	
Higher education	41 (6.6%)	18 (7.0%)	23 (6.3%)	
Population group				**<0.001**
General population	343 (55.1%)	113 (43.8%)	230 (63.2%)	
KPP*	279 (44.9%)	145 (56.2%)	134 (36.8%)	
Testing Modality				0.788
Outpatient, inpatient and TB clinics	325 (52.3%)	138 (53.5%)	187 (51.4%)	
VCT	94 (15.1%)	37 (14.3%)	57 (15.7%)	
ICT and SNS	110 (17.7%)	42 (16.3%)	68 (18.7%)	
Others**	93 (15.0%)	41 (15.9%)	52 (14.3%)	
Housing condition***				0.477
Stable	449 (72.9%)	182 (71.4%)	267 (74.0%)	
Unstable	167 (27.1%)	73 (28.6%)	94 (26.0%)	

Abbreviations: IQR, interquartile range; KPP, key and priority population; TB; tuberculosis; VCT, voluntarily counseling and testing; ICT, index case testing; SNS, social network strategy

KPP includes daily laborer (203; 72.8%), female sex worker (38; 13.6%), drivers (including long distance driver) (29;10.4%), military/police (7; 2.5%) and prisoner (2; 0.7%)

Bold significant  *p* < 0.05

**others include: community screening, private clinic, prevention of mother-to-child transmission clinic, sexually transmitted infection clinic, and family planning clinic, among others

***Participants with no permanent residence and those living with relatives or friends were categorized as having an unstable living situation, while those who owned or rented a house or apartment were categorized as having a stable living situation

### Recent infection among people with HIV newly enrolled to care

**Fig. 1:**
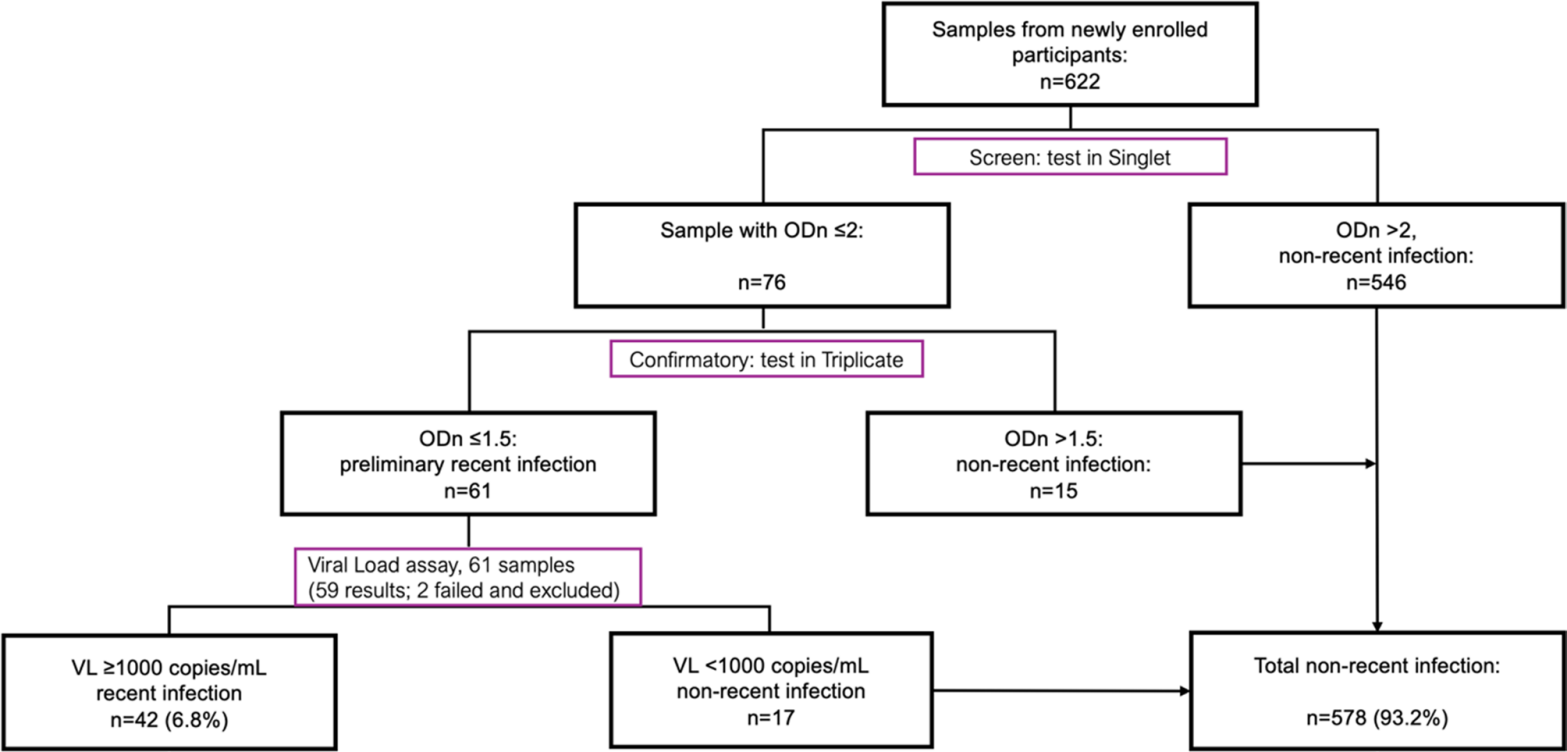
Flow diagram of the recent infection testing algorithm (RITA) for identifying recent HIV infections. Abbreviation: ODn, normalized optical density; VL, viral load

Of the 622 PLHIV newly enrolled to care, 61 (9.8%) participants had confirmatory ODn ≤ 1.5 in the LAg avidity assay, thus meeting the criteria for preliminary recent infection. Viral load was successfully determined for 59/61 cases with preliminary recent infection, while 2 failed and were excluded from further analysis. Among these 59, 42 (71.2%) had VL > 1000 copies/mL, while 17 (28.8%, 95% Wilson CI: 18.8% − 41.4%) had VL < 1000 copies/mL; hence, 42 participants (6.8%, [95% Wilson CI: 5.1% − 9.0%] of the study population) were classified as having recent infection (Fig. 1).

Among the 42 participants with recent infection, 23 (54.8%) had VL > 100,000 copies/mL. Interestingly, among these 23 individuals, 10 (43.5%) participants had VL > 1,000,000 copies/mL, and 5 of these exhibited very low ODn values of ≤0.4.

### Factors associated with recent infection among people with HIV newly enrolled to care

Participants with recent infection were younger (median 29 years) than those with non-recent infection (median 35 years); 14 (33.3%) of people with recent infection belonged to the youth age group compared to 62 (10.7%) of people with non-recent infection (*p* < 0.001). The distribution of socio-demographic and clinical characteristics of PLHIV newly enrolled in care with recent and non-recent HIV infection is presented in Table 2.

**Table 2:** Characteristics of PLHIV newly enrolled at urban ART clinics in central Ethiopia, by recency of HIV infection status

Characteristic	Recent infection (*n* = 42)	Non-recent infection (*n* = 578)			*p*-Value
Age (years; median, IQR)	29 (23–35)	35 (29–43)			
Age					**<0.001**
15–24 years	14 (33.3%)	62 (10.7%)			
≥25 years	28 (66.7%)	516 (89.3%)			
Sex					0.147
Male	13 (31.0%)	245 (42.4%)			
Female	29 (69.0%)	333 (57.6%)			
Population group					**0.021**
General Population	16 (38.1%)	326 (56.4%)			
KPP*	26 (61.9%)	252 (43.6%)			
Testing Modality					0.730
Outpatient, inpatient and TB clinics	20 (47.6%)	305 (52.8%)			
VCT	8 (19.0%)	85 (14.7%)			
ICT and SNS	9 (21.4%)	101 (17.5%)			
Others**	5 (11.9%)	87 (15.1%)			
Housing condition***					**0.018**
Stable	24 (57.1%)	423 (74.0%)			
Unstable	18 (42.9%)	149 (26.0%)			
Type of health facility					0.114
Health center	29 (69.0%)	327 (56.6%)			
Hospital	13 (31.0%)	251 (43.4%)			

Abbreviations: IQR, interquartile range; KPP, key and priority population; TB; tuberculosis; VCT, voluntarily counseling and testing; ICT, index case testing; SNS, social network strategy

*KPP includes female sex worker (*n* = 9), drivers (including long distance driver) (*n* = 2), prisoner(*n* = 0), military/police(*n* = 2) and daily laborer (*n* = 13); values represent recent infections only

**others include: community screening, private clinic, prevention of mother-to-child transmission clinic, sexually transmitted infection clinic, and family planning clinic, among others

***Participants with no permanent residence and those living with relatives or friends were categorized as having an unstable living situation, while those who owned or rented a house or apartment were categorized as having a stable living situation

**Table 3:** Univariate and multi-variable logistic regression analysis of factors associated with recent infection in PLHIV (≥15 years) newly enrolled in HIV care

Characteristic	Recent	Non-recent	COR (95% CI)	AOR (95% CI)
	n (%)	n (%)		
Age				
≥25 years	28 (66.7%)	516 (89.3%)	1	1
15–24 years	14 (33.3%)	62 (10.7%)	4.16 (2.08–8.33)	**4.25 (2.11–8.56)**
Sex				
Male	13 (31.0%)	245 (42.4%)	1	
Female	29 (69.0%)	333 (57.6%)	1.64 (0.84–3.22)	
Population group				
General Population	16 (38.1%)	326 (56.4%)	1	1
KPP*	26 (61.9%)	252 (43.6%)	2.10 (1.10–4.00)	**2.16 (1.12–4.15)**
Housing condition**				
Stable	24 (57.1%)	423 (74.0%)	1	
Unstable	18 (42.9%)	149 (26.0%)	2.13 (1.12–4.03)	

Abbreviations: COR, crude odds ratio; AOR, adjusted odds ratio; CI, confidence interval; KPP, key and priority populations

Bold AORs values indicate statistically significant associations

*KPP includes female sex worker, drivers (including long distance driver), prisoner, military/police and daily laborer

**Participants with no permanent residence and those living with relatives or friends were categorized as having an unstable living situation, while those who owned or rented a house or apartment were categorized as having a stable living situation

**Table 4:** Sex-stratified logistic regression analysis of factors associated with recent infection

Characteristic	Recent	Non-recent	COR (95% CI)	AOR (95% CI)
	Number (%)	Number (%)		
Female (Recent infection 29, non-recent infection, 333)				
Age				
≥25 years	18 (62.1%)	282 (84.7%)	1	1
15–24 years	11 (37.9%)	51 (15.3%)	3.38 (1.51–7.58)	**2.66 (1.15–6.14)**
Population group				
General Population	12 (41.4%)	217 (65.2%)	1	1
KPP*	17 (58.6%)	116 (34.8%)	2.65 (1.22–5.74)	**2.35 (1.06–5.20)**
Housing condition**				
Stable	14 (48.3%)	251 (76.1%)	1	1
Unstable	15 (51.7%)	79 (23.9%)	3.40 (1.58–7.36)	**2.90 (1.31–6.41)**
Male (Recent infection 13, non-recent infection, 245)				
Age				NA
≥25 years	10 (76.9%)	234 (95.5%)	1	
15–24 years	3 (23.1%)	11 (4.5%)	6.38 (1.54–26.53)	
Population group				NA
General Population	4 (30.8%)	109 (44.5%)	1	
KPP*	9 (69.2%)	136 (55.5%)	1.80 (0.54–6.01)	
Housing condition**				NA
Stable	10 (76.9%)	172 (71.1%)	1	
Unstable	3 (23.1%)	70 (28.9%)	0.74 (0.20–2.76)	

Abbreviations: COR, crude odds ratio; AOR, adjusted odds ratio; CI, confidence interval; KPP, key and priority population

Bold AORs values indicate statistically significant associations

*KPP includes female sex worker, drivers (including long distance driver), prisoner, military/police and daily laborer

***Participants with no permanent residence and those living with relatives or friends were categorized as having an unstable living situation, while those who owned or rented a house or apartment were categorized as having a stable living situation

In univariable analysis of associations with recent infection, age, sex, population group, and housing condition had *p*-values < 0.25. Following stepwise removal of variables with *p* > 0.05, youth age group (adjusted odds ratio (aOR) 4.25, 95% CI, 2.11–8.56) and KPP group (aOR, 2.16; 95%CI, 1.12–4.15) remained significantly associated with recent infection (Table 3). Effect modification by sex was assessed using interaction terms between sex and the other variables (age, population group, and housing condition, respectively) and we found a suggestive (borderline) effect modification for sex*housing condition, *p* = 0.067. This was further investigated in sex-stratified analyses. Among female participants, youth age group (aOR, 2.66; 95%CI, 1.15–6.14), KPP group (aOR, 2.35; 95%CI, 1.06–5.20), and unstable housing condition (aOR, 2.90; 95%CI, 1.31–6.41) remained statistically significant. Among male participants, youth age group was the only factor with *p* < 0.25 in univariable analysis, and it was significantly associated with recent HIV infection (Table 4)

## Discussion

In this study, based on participants of a cohort study performed in an uptake area in Ethiopia with high HIV prevalence, we found a relatively low proportion of recent HIV infection among PLHIV newly enrolled to care in public health facilities. Youth and persons belonging to KPP had higher odds of having recent infection at the time of enrollment to care. However, in sex-stratified analyses, belonging to KPP was associated with recent HIV infection only among women.

Considering progress of HIV/ART programs in the East African region over the last decades, it is important to understand and characterize people with recently acquired HIV infection in order to improve and optimize the test-and-treat strategy, and to design tailored HIV prevention methods. The most accurate way for identifying recent HIV infection relies on following individuals at risk of acquiring HIV infection at frequent intervals; however, this approach is not feasible outside research settings. The use of alternative methods based on cross-sectional data, including serological and molecular methods, offers possibilities to estimate recency and has increased in recent years [8, 9, 24, 25]. For example, time since infection can be estimated by assessing intra-host viral diversity, which evolves over time after HIV acquisition, to estimate time since infection [8, 24]. Serological assays are commonly used to identify recent infection, especially for surveillance, as these methods are rapid, less expensive and do not require advanced infrastructure [8, 25].

The LAg-Avidity assay is a serologic test with improved precision in categorizing individuals as being recently infected or not, by measurement of relative antigen binding strength (avidity), which increases over the course of infection [26]. Thus, this assay reflects the functional property of maturing antibodies, and uses recombinant proteins that cover the immunodominant region of HIV gp41 from all major subtypes and recombinants of HIV-1 group M [27, 28]. Since serologic tests can overestimate recency, WHO recommends RITA, which uses both a serological assay and VL to minimize such misclassification [9]. Currently, the two main contexts for use of HIV recency testing are incidence estimation at population level and research studies, but it has also been introduced in some routine care settings to identify recent infection among newly HIV diagnosed persons as part of case-based surveillance [13, 14, 29].

In our study, the proportion of recent HIV infection was 6.8%, which is lower than the 14.2% and 11.3% reported by two previous studies from Ethiopia [13, 14]. While the effect of regional differences within the country cannot be entirely ruled out, the key difference is that these studies relied only on serologic assays to classify participants as being recently infected, whereas we incorporated VL into our criteria. Similar to our findings of 28.8% (17/59), previous studies from other sub-Saharan African countries (Kenya, Rwanda, Lesotho and Malawi) found that around 20%-50% of infections classified as recent by serologic assay had suppressed VL [20, 29–31]. This highlights the importance of incorporating VL in RITA to reduce false positive results on the HIV recency assay [9]. Suppressed VL among people newly enrolling to care implies undisclosed ART use (‘silent transfer’), and has been reported in clinical trial settings [32] and community surveys [33, 34], but it has not been well studied in routine clinical settings.

More than half of the participants with recent infection had VL > 100,000 copies/mL including 10 participants with VL > 1,000,000 copies/mL. Interestingly, a high proportion of those had lower avidity antibodies and reported symptoms such as fever, fatigue and night sweating, which could suggest symptomatic primary HIV infection [35]. Studies from different settings have shown that people with recent HIV infection, especially those at early stage of infection, contribute disproportionately to onward HIV transmission [4, 5, 36, 37]. Hence, determining recency of HIV infection as part of routine care could be an important tool to guide targeted and intensified interventions, including thorough and systematic partner notification and testing (contact tracing), investigating transmission networks, as well as early ART initiation to reduce the risk of onward transmission and prevent new HIV infections.

Among our study participants, a higher proportion of recent infection at enrollment to care was observed among youth. This association remained significant in both male and female participants in sex-stratified analysis. Other studies from similar settings also reported higher odds of recent infection among adolescent and young adults [14, 29, 30, 38]. Our finding likely reflects both disproportionately high rates of new HIV infections among adolescents (particularly females) and the impact of tailored interventions and initiatives such as ‘All In to end adolescent AIDS’, which may have improved early case detection [12, 39].

Apart from the association with age group, persons defined as belonging to KPP were more likely to have recent infection at enrolment to care. However, sex-stratified analysis revealed that this association was significant only for female participants, suggesting that the observed relationship in the overall model may be largely driven by female subgroup. Notably, nearly one third of female participants with recent infection were CSWs, a finding which could reflect effectiveness of CSW-focused interventions in achieving early HIV detection among this high-risk group.

Overall, limited data availability and variations in the definitions of key and priority population across different settings and studies make it difficult to compare the prevalence of new HIV infections among KPP newly enrolled to care. For example, men who have sex with men and people who inject drugs are not included in the list of KPP identified by the Ethiopian national roadmap for HIV prevention and HIV/AIDS national strategic plan [12]. However, KPP are consistently reported to be at higher risk of acquiring HIV, and thereby contribute to onward transmission [40, 41]. The UNAIDS 2024 report which investigated the proportion of new HIV infections among key populations found that 55% of all new HIV infections in 2022 occurred in such groups [41]. In agreement with this, nearly half (44.7%) of PLHIV newly enrolled at our study sites and the majority (61.9%) of persons with recent infections in our study belonged to KPP. Our finding aligns with and strengthens the existing rationale for prioritizing interventions targeting KPP to control HIV transmission and prevent new infections.

While no significant association between housing condition and recent HIV infection was observed in the overall (pooled) model, except for borderline interaction with sex (*p* = 0.067), sex-stratified analysis revealed statistically significant higher odds of recent infection among female participants with unstable housing condition. This suggests that the effect of unstable housing condition is modified by sex, and that the overall model masked this association, which could have programmatic and policy implications. The underlying reason for the association between unstable housing condition and recent infection among females is not clear; possibly, this could be due to undisclosed sex work (survival sex) in economically disadvantaged women [42–44].

The higher proportion of recent infection among youth and among women belonging to KPP may indicate high rate of transmission among these groups. Yet, it can also reflect the effectiveness of targeted interventions for female CSW that allowed for detection of recently acquired HIV infection. Furthermore, our findings imply that similar targeted interventions should be considered for other KPP categories, in particular adapted to men, who are at high risk of late presentation to HIV care.

Overall, since our study focused on PLHIV newly enrolled to care, the observed low proportion of recent infection indicates late diagnosis rather than low transmission, a finding in line with data from several studies showing that late presentation to care remains common in this region [45–47]. Preliminary results from the parent cohort (THESA) show that almost half of PLHIV presented with advanced HIV disease at enrollment to care [48]. Hence, there is a need for identifying barriers to early care and implementing targeted interventions, for example self-testing and mobile outreach for improved early identification of PLHIV in this setting [49].

To the best of our knowledge, this is the first study in Ethiopia using the combination of serology and VL as a criterion to identify recent HIV infections. This reduces misclassification and improves the accuracy of categorizing cases as recent or non-recent infections. Our study has some limitations. First, we determined recency of HIV infection among PLHIV newly enrolled to HIV care; however, such persons might differ from people diagnosed with HIV but not linked to care. Hence, our findings may not be generalizable to all individuals newly diagnosed with HIV. Second, the relatively small number of male participants with recent infection included in this study limits the statistical power of stratified analyses. Third, HIV diagnosis, as well as identification of recent HIV infection, was based on antibody tests; thus, acute HIV infections may be missed, potentially leading to underestimation of recent infections. Fourth, the use of undisclosed ART could potentially have impacted our findings. Data on antiretroviral drug metabolites may improve accuracy of determining recent infection, which was not part of our study protocol [50]. However, our study protocol included detailed questions on ART history, and persons diagnosed with HIV for more than a week at the time of enrolment to care were not included in the current study. Finally, data on ART use for pre-or post-exposure prophylaxis was not available. Since such prophylactic treatment could affect antibody maturation (and thus diagnosis of recent infection), future studies in this field may need to incorporate these variables [51].

## Conclusions

A relatively low proportion PLHIV newly enrolled to care in this uptake area in central Ethiopia had recent HIV infection. This is likely to contribute to delayed interventions for PLHIV, allowing for uncontrolled onward transmission. Hence, there is a need for improved HIV testing programs for more timely identification of PLHIV in this setting. Moreover, a higher proportion of recent infection was observed among youth and females belonging to key and priority populations. This finding could indicate that recent HIV transmissions are concentrated in the identified sub-groups in the uptake area but may also reflect the effectiveness of interventions targeted to these groups.

## Data Availability

The data that support the findings of this study are available from the corresponding author upon reasonable request.

## Abbreviations

ART: Antiretroviral therapy
CSW: Commercial sex worker
KPP: Key and priority populations
Lag: Limiting antigen
ODn: Normalized optical density
PLHIV: People living with HIV
RITA: Recent infection testing algorithm
THESA: Transmission of HIV in the era of scaling up access to antiretroviral therapy
VL: Viral load
WHO: World health organization

## References

[1] Joint United Nations Programme on HIV/AIDS. (UNAIDS). PATH THAT ENDS AIDS, 2023 UNAIDS Global AIDS Update. 2023. Report No.

[2] World Health Organization. HIV statistics, globally and by WHO region, UNAIDS/WHO estimates, 2024. [Internet]. Available from: https://cdn.who.int/media/docs/default-source/hq-hiv-hepatitis-and-stis-library/j0482-who-ias-hiv-statistics_aw-1_final_ys.pdf?sfvrsn=61d39578_3.

[3] Consolidated Guidelines on HIV Prevention. Testing, treatment, service delivery and monitoring: recommendations for a Public Health approach. 1st. Geneva: World Health Organization; 2021. p. 1 p. 34370423

[4] Marzel A, Shilaih M, Yang WL, Böni J, Yerly S, Klimkait T, et al. HIV-1 transmission During recent infection and During treatment interruptions as Major drivers of New infections in the Swiss HIV cohort study. Clin Infect Dis. 2016, Jan, 1;62(1):115–22. 10.1093/cid/civ732. 26387084 10.1093/cid/civ732

[5] Pilcher CD, Tien H, Eron, Jr. JJJ, Vernazza PL, Leu S, Stewart PW, et al. Brief but efficient: acute HIV infection and the sexual transmission of HIV. The J Infect Dis. 2004, May, 15;189(10):1785–92. 10.1086/386333. 15122514 10.1086/386333

[6] Eaton LA, Kalichman SC. Changes in transmission risk behaviors across stages of HIV disease among people living with HIV. J Assoc Nurses AIDS Care. 2009, Jan;20(1):39–49. 10.1016/j.jana.2008.10.005. 19118770 10.1016/j.jana.2008.10.005 PMC3560412

[7] Colfax GN, Buchbinder SP, Cornelisse PGA, Vittinghoff E, Mayer K, Celum C. Sexual risk behaviors and implications for secondary HIV transmission during and after HIV seroconversion. AIDS. 2002, Jul;16(11):1529–35. 10.1097/00002030-200207260-00010. 12131191 10.1097/00002030-200207260-00010

[8] Moyo S, Wilkinson E, Novitsky V, Vandormael A, Gaseitsiwe S, Essex M, et al. Identifying recent HIV infections: from serological assays to genomics. Viruses. 2015, Oct, 23;7(10):5508–24. 10.3390/v7102887. 26512688 10.3390/v7102887 PMC4632395

[9] Using recency assays for HIV surveillance using recency assays for HIV surveillance: 2022 technical guidance. Geneva: Joint U Nations Programme HIV/AIDS The World Health Organ. 2022. Licence: CC BY-NC-SA 3.0 IGO.

[10] Laeyendecker O, Brookmeyer R, Oliver AE, Mullis CE, Eaton KP, Mueller AC, et al. Factors associated with incorrect identification of recent HIV infection using the BED capture Immunoassay. Aids Res Hum Retrov. 2012, Aug;28(8):816–22. 10.1089/aid.2011.0258. 10.1089/aid.2011.0258 PMC3399553 22014036

[11] The Ethiopian Public Health Institute. HIV related Estimates and projections in Ethiopia for the Year 2024–2025 June 2025, Addis Ababa Ethiopia. https://ephi.gov.et/wp-content/uploads/2025/08/Ethiopian-HIV-Estimates-and-projecti-for-the-year-2024-and-2025-EPHI-Augst142025.pdf.

[12] Ministry of Health, Ethiopia. HIV/AIDS national strategic plan 2023/24 - 2026/27. 2023.

[13] Alemu T, Ayalew M, Haile M, Amsalu A, Ayal A, Wale F, et al. Recent HIV infection among newly diagnosed cases and associated factors in the Amhara regional state, Northern Ethiopia: HIV case surveillance data analysis (2019-2021). Front Public Health. 2022, Nov;10(10):922385. 10.3389/fpubh.2022.922385. 36457319 10.3389/fpubh.2022.922385 PMC9706085

[14] Admasu N, Lomboro A, Kebede E, Bejiga B, Bulti J, Abdella S, et al. Recent HIV infection and associated factors among newly diagnosed HIV cases in the Southwest Ethiopia regional state: HIV case-based surveillance analysis (2019–2022). BMC Infect Dis. 2024, Jun, 20;24(1):609. 10.1186/s12879-024-09481-z. 38902626 10.1186/s12879-024-09481-z PMC11188228

[15] Biomedical M. Inc. Maxim HIV-1 Limiting antigen Avidity EIA Single well Avidity enzyme Immunoassay for detection of recent HIV-1 infection [Internet]. 2022. Available from: https://maximbio.com/wp-content/uploads/920013-Maxim-HIV-1-LAg-Avidity-EIA-Product-Insert-LN23015.06-1.pdf.

[16] PCR Biosystems Ltd. qPCRBIO probe 1-step Virus Detect.

[17] Kuhlmann AS, Haworth KG, Barber-Axthelm IM, Ironside C, Giese MA, Peterson CW, et al. Long-Term persistence of anti-HIV broadly neutralizing Antibody-secreting hematopoietic cells in humanized mice. Mol Ther. 2019, Jan;27(1):164–77. 10.1016/j.ymthe.2018.09.017. 30391142 10.1016/j.ymthe.2018.09.017 PMC6318702

[18] Cobas® HIV-1. Quantitative nucleic acid test for use on the cobas® 5800/6800/8800 systems for in vitro diagnostic use.

[19] Kassanjee R, Pilcher CD, Busch MP, Murphy G, Facente SN, Keating SM, et al. Viral load criteria and threshold optimization to improve HIV incidence assay characteristics. AIDS. 2016, Sep, 24;30(15):2361–71. 10.1097/QAD.0000000000001209. 27454561 10.1097/QAD.0000000000001209 PMC5024345

[20] Telford CT, Tessema Z, Msukwa M, Arons MM, Theu J, Bangara FF, et al. Geospatial transmission hotspots of recent HIV infection — Malawi, October 2019–March 2020, October 2019-March 2020. MMWR Morb. Mortal. Wkly. Rep. 71(9):329–34. 10.15585/mmwr.mm7109a1. 2022 Mar 4. 10.15585/mmwr.mm7109a1 PMC8893337 35239633

[21] Sayyah M, Rahim F, Kayedani GA, Shirbandi K, Saki-Malehi A. Global view of HIV prevalence in prisons: a systematic Review and meta-analysis. ijph. 2019, May, 8. 10.18502/ijph.v48i2.816. PMC6556176 31205875

[22] Ba O, O’Regan C, Nachega J, Cooper C, Anema A, Rachlis B, et al. HIV/AIDS in African militaries: an ecological analysis. Med Confl Surviv. 2008, Apr;24(2):88–100. 10.1080/13623690801950260. 18488671 10.1080/13623690801950260

[23] Consolidated Guidelines on HIV. Viral hepatitis and STI prevention, diagnosis, treatment and care for key populations. 1st. Geneva: World Health Organization; 2022. p. 1 p. 36417550

[24] Puller V, Neher R, Albert J. Estimating time of HIV-1 infection from next-generation sequence diversity. PLoS Comput Biol. 2017, Oct, 2;13(10):e1005775. 10.1371/journal.pcbi.1005775. Wilke, CO, editor.. 10.1371/journal.pcbi.1005775 PMC5638550 28968389

[25] Facente SN, Grebe E, Maher AD, Fox D, Scheer S, Mahy M, et al. Use of HIV recency assays for HIV incidence estimation and other surveillance use cases: systematic Review. JMIR Public Health Surveill. 2022, Mar, 11;8(3):e34410. 10.2196/34410. 10.2196/34410 PMC8956992 35275085

[26] Chawla A, Murphy G, Donnelly C, Booth CL, Johnson M, Parry JV, et al. Human Immunodeficiency Virus (HIV) Antibody Avidity testing to identify recent infection in newly diagnosed HIV type 1 (HIV-1)-seropositive persons infected with diverse HIV-1 subtypes. J Clin Microbiol. 2007, Feb;45(2):415–20. 10.1128/JCM.01879-06. 17151211 10.1128/JCM.01879-06 PMC1829080

[27] Wei X, Liu X, Dobbs T, Kuehl D, Nkengasong JN, Hu DJ, et al. Development of two Avidity-based assays to Detect recent HIV type 1 seroconversion using a multisubtype gp41 recombinant protein. Aids Res Hum Retrov. 2010, Jan;26(1):61–71. 10.1089/aid.2009.0133. 10.1089/aid.2009.0133 20063992

[28] Duong YT, Qiu M, De AK, Jackson K, Dobbs T, Kim AA, et al. Detection of recent HIV-1 infection using a new Limiting-antigen Avidity assay: potential for HIV-1 incidence Estimates and Avidity maturation studies. PLoS One. 2012, Mar, 27;7(3):e33328. 10.1371/journal.pone.0033328. Landay, A, editor.. 10.1371/journal.pone.0033328 PMC3314002 22479384

[29] Welty S, Motoku J, Muriithi C, Rice B, de Wit M, Ashanda B, et al. Brief report: recent HIV infection surveillance in routine HIV testing in Nairobi, Kenya: a Feasibility study. JAIDS J Acquired Immune Deficiency Syndromes. 2020;84(1):5–9. 10.1097/QAI.0000000000002317. 10.1097/QAI.0000000000002317 32058458

[30] Rwibasira GN, Malamba SS, Musengimana G, Nkunda RCM, Omolo J, Remera E, et al. Recent infections among individuals with a new HIV diagnosis in Rwanda, 2018–2020. PLoS One. 2021, Nov, 17;16(11):e0259708. 10.1371/journal.pone.0259708. Lu, X, editor.. 10.1371/journal.pone.0259708 PMC8598012 34788323

[31] Mohloanyane T, Olivier D, Labhardt ND, Amstutz A. Recent HIV infections among newly diagnosed individuals living with HIV in rural Lesotho: secondary data from the VIBRA cluster-randomized trial. PLoS One. 2022, Nov, 21;17(11):e0277812. 10.1371/journal.pone.0277812. Lessells, RJ, editor.. 10.1371/journal.pone.0277812 PMC9678280 36409754

[32] Marzinke MA, Clarke W, Wang L, Cummings V, Liu TY, Piwowar-Manning E, et al. Nondisclosure of HIV status in a Clinical trial setting: Antiretroviral Drug screening can help distinguish between newly diagnosed and Previously diagnosed HIV infection. Clin Infect Dis. 2014, Jan, 1;58(1):117–20. 10.1093/cid/cit672. 24092804 10.1093/cid/cit672 PMC3864502

[33] Manne-Goehler J, Rohr J, Montana L, Siedner M, Harling G, Gómez-Olivé FX, et al. ART denial: results of a home-based study to Validate self-reported antiretroviral use in Rural South Africa. AIDS Behav. 2019, Aug;23(8):2072–78. 10.1007/s10461-018-2351-7. 30523490 10.1007/s10461-018-2351-7 PMC6551321

[34] Kim AA, Mukui I, Young PW, Mirjahangir J, Mwanyumba S, Wamicwe J, et al. Undisclosed HIV infection and antiretroviral therapy use in the Kenya AIDS indicator survey 2012: relevance to national targets for HIV diagnosis and treatment. AIDS. 2016, Nov, 13;30(17):2685–95. 10.1097/QAD.0000000000001227. 27782965 10.1097/QAD.0000000000001227 PMC6559732

[35] Quinn TC, Wawer MJ, Sewankambo N, Serwadda D, Li C, Wabwire-Mangen F, et al. Viral load and Heterosexual transmission of Human Immunodeficiency Virus type 1. N Engl J Med. 2000, Mar, 30;342(13):921–29. 10.1056/NEJM200003303421303. 10738050 10.1056/NEJM200003303421303

[36] Boily MC, Baggaley RF, Wang L, Masse B, White RG, Hayes RJ, et al. Heterosexual risk of HIV-1 infection per sexual act: systematic review and meta-analysis of observational studies. The Lancet Infect Dis. 2009, Feb;9(2):118–29. 10.1016/S1473-3099(09)70021-0. 19179227 10.1016/S1473-3099(09)70021-0 PMC4467783

[37] Pinkerton SD. Probability of HIV transmission During Acute infection in Rakai, Uganda. AIDS Behav. 2008, Sep;12(5):677–84. 10.1007/s10461-007-9329-1. 18064559 10.1007/s10461-007-9329-1 PMC2614120

[38] Currie DW, West CA, Patel HK, Favaloro J, Asiimwe M, Ndagije F, et al. Risk factors for recent HIV infections among adults in 14 countries in Africa identified by population-based HIV Impact assessment surveys, 2015–2019. Emerg Infect Dis. 2023, Nov;29(11). 10.3201/eid2911.230703. 10.3201/eid2911.230703 PMC10617335 37877591

[39] Joint United Nations Programme on HIV/AIDS (UNAIDS). United Nations Children’s fund (UNICEF). ALL IN to end the adolescent AIDS epidemic - a progress report. 2016. Geneva: UNAIDS.

[40] Jin H, Restar A, Beyrer C. Overview of the epidemiological conditions of HIV among key populations in Africa. J Int AIDS Soc. 2021, Jul;24(S3):e25716. 10.1002/jia2.25716. 10.1002/jia2.25716 PMC8242974 34190412

[41] New HIV infections among key populations, proportions in 2010 and 2022. Licence: cC BY-NC-SA 3.0. Geneva: Joint United Nations Programme on HIV/AIDS IGO; 2023.

[42] International Labour Office. HIV/AIDS and poverty - the critical connection. ILO Programme HIV/AIDS The World Work https://Www.Ilo.org/Aids. 2005, Oct.

[43] Baru A, Adeoye IA, Adekunle AO. “I was raped by the broker on the first day of my arrival in the town.” exploring reasons for risky sexual behavior among sexually-active unmarried young female internal migrants in Ethiopia: a qualitative study. In: Wasserman D, editor. PLoS ONE. Vol. 15(11). 2020 Nov 13. p. e0242176. 10.1371/journal.pone.0242176. 10.1371/journal.pone.0242176 PMC7665896 33186376

[44] Chimdessa A, Cheire A. Sexual and physical abuse and its determinants among street children in Addis Ababa, Ethiopia 2016. BMC Pediatr. 2018, Dec;18(1):304. 10.1186/s12887-018-1267-8. 30231892 10.1186/s12887-018-1267-8 PMC6146752

[45] Ainembabazi B, Katana E, Bongomin F, Wanduru P, Mayega RW, Mukose AD. Prevalence of advanced HIV disease and associated factors among antiretroviral therapy naïve adults enrolling in care at public health facilities in Kampala, Uganda. Ther Adv Infect. 2024, Jan;11:20499361241251936. 10.1177/20499361241251936. 10.1177/20499361241251936 PMC11103927 38770168

[46] Carmona S, Bor J, Nattey C, Maughan-Brown B, Maskew M, Fox MP, et al. Persistent high burden of advanced HIV disease among patients seeking care in South Africa’s National HIV program: data from a nationwide laboratory cohort. Clin Infect Dis. 2018, Mar;66;66(suppl_2):S111–7. 10.1093/cid/ciy045. 10.1093/cid/ciy045 PMC5850436 29514238

[47] Ford N, Kassanjee R, Stelzle D, Jarvis JN, Sued O, Perrin G, et al. Global prevalence of advanced HIV disease in healthcare settings: a rapid review. J Int AIDS Soc. 2025, Feb;28(2):e26415. 10.1002/jia2.26415. 10.1002/jia2.26415 PMC11802239 39915008

[48] Abdulahi IJ, Björkman P, Mulu A, Abdissa A, Medstrand P, Reepalu A. Advanced HIV disease remains common among people with HIV newly enrolled to care in central Ethiopia in the test-and-treat Era. in: https://i-base.info/htb/52309.

[49] Hatzold K, Gudukeya S, Mutseta MN, Chilongosi R, Nalubamba M, Nkhoma C, et al. HIV self-testing: breaking the barriers to uptake of testing among men and adolescents in sub-Saharan Africa, experiences from STAR demonstration projects in Malawi, Zambia and Zimbabwe. J Intern AIDS Soc. 2019, Mar;22;Suppl 22(S1):e25244. 10.1002/jia2.25244 PubMed PMID: 30907505; PubMed Central PMCID: PMC6432104. 10.1002/jia2.25244 PMC6432104 30907505

[50] University, Lund HIV Transmission in the Era of Scaling up Access to Antiretroviral Therapy in Ethiopia [Clinical trial registration] [Internet]. clinicaltrials.gov; Report No.: nCT05652400. Available from: Mar.2025. https://clinicaltrials.gov/study/NCT05652400. 2025 Nov 12.

[51] Parker I, Khalil G, Martin A, Martin M, Vanichseni S, Leelawiwat W, et al. Altered Antibody responses in persons infected with HIV-1 while using preexposure prophylaxis. Aids Res Hum Retrov. 2021, Mar, 1;37(3):189–95. 10.1089/aid.2020.0137 PubMed PMID: 33126825; PubMed Central PMCID: PMC8020499. 10.1089/aid.2020.0137 PMC8020499 33126825

[52] National guideline for comprehensive HIV prevention, care and treatment, Ministry of health, Ethiopia. 2022 February.

